# *Lagotis brachystachya* maxim attenuates chronic alcoholic liver injury combined with gouty arthritis in rats *via* its anti-inflammatory activity

**DOI:** 10.3389/fphar.2022.995777

**Published:** 2022-09-13

**Authors:** Min-Xia Guo, Man-Man Zhang, Hai-Yan Yang, Chu-Ling Zhang, Hong-Yu Cheng, Na-Zhi Li, Li-Tao Yi, Ji-Xiao Zhu

**Affiliations:** ^1^ Research Center of Natural Resources of Chinese Medicinal Materials and Ethnic Medicine, Jiangxi University of Chinese Medicine, Nanchang, China; ^2^ Department of Chemical and Pharmaceutical Engineering, Huaqiao University, Xiamen, China; ^3^ College of Humanities, Jiangxi University of Chinese Medicine, Nanchang, China

**Keywords:** Lagotis brachystachya maxim, alcoholic liver injury, gouty arthritis, inflammation, TLR4, NLRP3, stat3

## Abstract

*Lagotis brachystachya* Maxim, a common herb in Tibetan medicine, is mainly used to treat pneumonia, hepatitis, yellow water disease (gouty arthritis). Since long-term heavy drinking is also a risk factor for gouty arthritis, the present study aimed to evaluate the underlying protective role and mechanism of extracts of *Lagotis brachystachya* (ELB) in chronic alcoholic liver injury combined with gouty arthritis. The rat chronic alcoholic liver injury combined with gouty arthritis model was established by long-term alcohol consumption and monosodium urate (MSU) injection. The therapeutical action of ELB was then evaluated by biochemical measurement, histopathological examination, ankle swelling assessment, and protein detection. According to biochemical measurements and histopathological evaluation, ELB could alleviate the symptoms of alcoholic liver injury combined with gouty arthritis. In addition, chronic alcohol consumption and MSU activated inflammatory-related signaling such as TLR4/MyD88/NF-κB, NLRP3, and JAK2/STAT3 pathways in the liver and synovial tissues, while ELB significantly inhibited the activation of the inflammatory signaling pathway. In conclusion, ELB is protective in rats with chronic alcoholic liver injury and gouty arthritis, possibly mediated by the inhibition of TLR4/MyD88/NF-κB, NLRP3, and JAK2-STAT3 signaling pathways in both the hepatic and synovial tissues.

## Introduction

The alcoholic liver injury, which initially manifests as alcoholic fatty liver disease, is primarily caused by heavy or long-term alcohol consumption. It can further develop into alcoholic hepatitis, liver fibrosis, cirrhosis, or even liver cancer (National Workshop on Fatty Liver and Alcoholic Liver Disease, 2018). It is estimated that approximately 20% of the drinking population worldwide has varying degrees of alcoholic liver disease. Alcoholic cirrhosis-induced death accounts for 48% of the mortality in patients with liver cirrhosis (Zhang, 2013). Alcoholic liver injury, the second-largest liver disease after viral hepatitis, has become one of China’s most important chronic liver diseases (Xu et al., 2003; Chang et al., 2022).

Besides alcoholic liver injury, long-term heavy drinking is also a risk factor for gouty arthritis. Generally, gouty arthritis is mainly caused by increased serum uric acid levels, leading to the deposition of urate crystals at the joints in patients. The deposited crystals trigger the release of inflammatory mediators and other cytokines in the human body, which cause inflammatory reactions in the joints of patients (George and Simkin, 2006). On the other hand, the malt component within the alcohol contains high purine content. Therefore, heavy drinking will provide excessive exogenous purines and thus increase uric acid levels in the body (Liu Y. et al., 2021), which induces the onset of gouty arthritis.

The malt in alcohol is a food with a high purine content, which means alcohol drinking will result in excessive uric acid in the body (Liu Y. et al., 2021), inducing the onset of gouty arthritis. The inflammatory reactions caused by these two diseases are related to the similar inflammatory signaling pathways mediated by inflammatory factors such as TNF-α, IL-1β, and IL-6, as well as the inflammatory-related signaling pathway such as NF-κB, NLRP3, and JAK2-STAT3 (Dunn and Shah, 2016; Galozzi et al., 2021).

*Lagotis brachystachya* Maxim is a common characteristic medicinal material in Tibetan medicine (tashi, 2006). It is mainly used to treat pneumonia, hepatitis, and Yellow Water Disease in the local Tibet area (poncuo, 2012). According to traditional Chinese Medicine and Tibetan medicine theories, Yellow Water Disease is one form of arthritis (BianBa et al., 2019). Our previous studies have shown that extracts of *Lagotis brachystachya* (ELB) exerted therapeutic effects on inflammatory regulation (Shan et al., 2020a; Shan et al., 2020b; Shan et al., 2021). Since inflammation is one of the most common features of liver injury and gouty arthritis, we hypothesized that ELB could exert protective actions against liver injury and gouty arthritis. Therefore, in the present study, the underlying protective role and mechanism of ELB involved in TLR4/NF-κB, NLRP3, and JAK2/STAT3 inflammatory signaling pathways were investigated in chronic alcoholic liver injury combined with gouty arthritis.

## Materials and methods

### Animals

Male SPF SD rats (140–170 g) were purchased from the Animal Center of Jiangxi University of Traditional Chinese Medicine [Certificate: SCXK (Jiangxi) 2018–0003], and raised under the conditions of temperature at (22 ± 2) °C, relative humidity at 60 ± 5%. The whole experimental protocol was reviewed and approved by the animal committee of the Jiangxi University of Chinese Medicine (JZLLSC 2019–0221). The animals had free access to water and food during the experimental procedure.

### Drugs and reagents

*Lagos brachystachya* Maxim was collected in Jilong of Tibet province, China on 08/10/2019. It was identified as a dried whole *Lagotis brachystachya* of Scrophulariaceae by Associate Professor Du Xiaolang, Research Center of Chinese Medicinal Resources and Ethnic Medicine, Jiangxi University of Chinese Medicine. A voucher specimen (No. 09-05-07-19) was deposited at the Research Center of Chinese Medicinal Resources and Ethnic Medicine. Edible alcohol was purchased from Beijing Hong-Xing Co., Ltd. (Beijing, China). Bifendate were purchased from Million Bond Pharmaceutical Group Co., Ltd. (Zhejiang, China). Colchicine was purchased from Xishuangbanna Pharmaceutical Co., Ltd. (Yunnan, China). Aspartate aminotransferase (AST), alanine aminotransferase (ALT), uric acid (UA), triglyceride (TG), serum total cholesterol (TC), xanthine oxidase (XOD), total bilirubin kit (TBIL), alkaline phosphatase (ALP), malondialdehyde (MDA) and superoxide dismutase (SOD) kits were purchased from Nanjing Jiancheng bioengineering Institute (Nanjing, China). Tumor necrosis factor-α (TNF-α), interleukin-6 (IL-6), and interleukin-1β (IL-1β) ELISA kits were purchased from CUSABIO BIOTECH CO, Ltd. (Wuhan, China). Anti-β-actin (D6A8) antibody was purchased from Cell Signaling Technology (Beverly, United States). The anti-TLR4 antibody, anti-pNF-κB antibody, anti-NLRP3 antibody, anti-Caspase-1 antibody, anti-MyD88 antibody, anti-pJAK2 antibody, and anti-pSTAT3 antibody were purchased from Abcam (Cambridge, United Kingdom).

### Preparation and component analysis of extracts of *Lagotis brachystachya*

**FIGURE 1:**
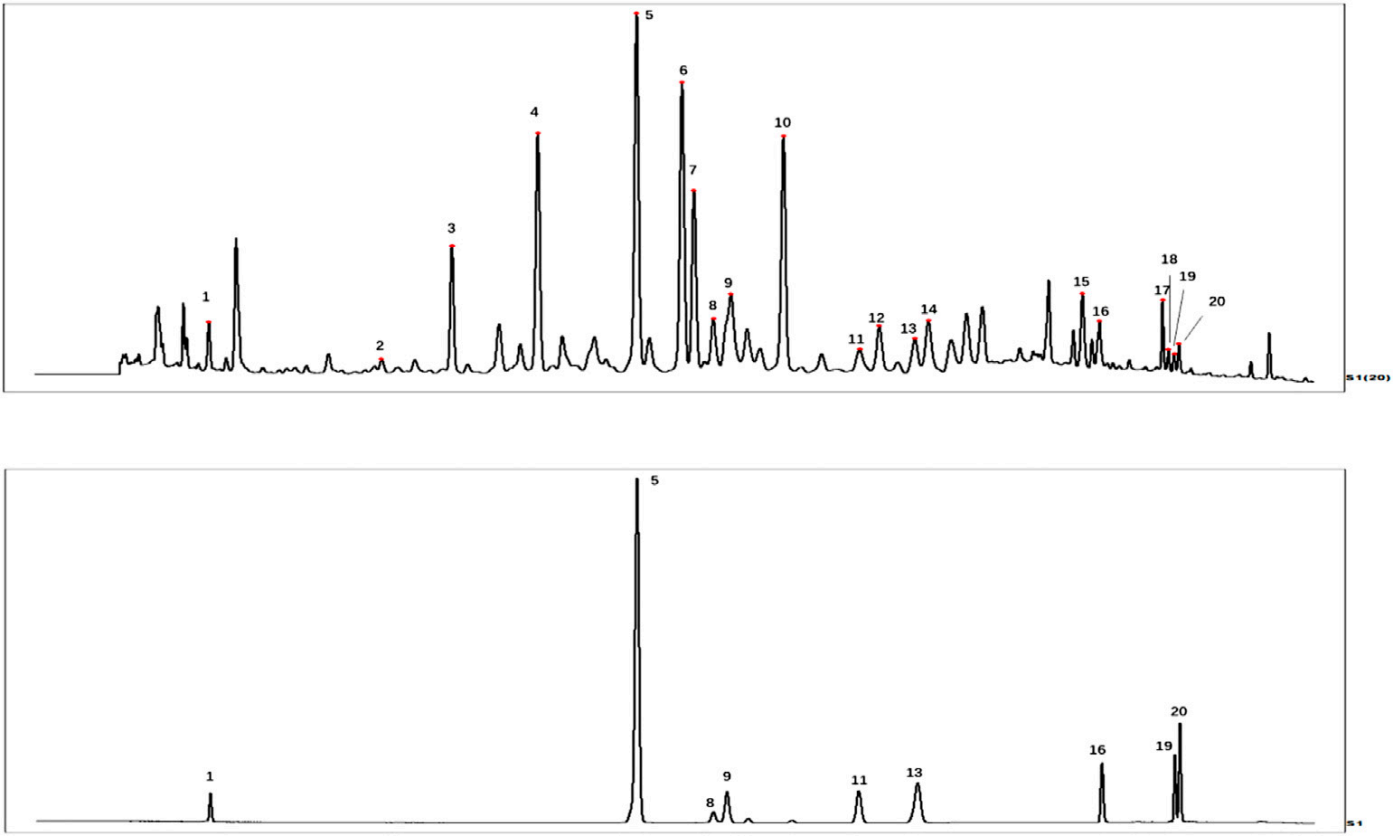
HPLC fingerprint of ELB **(A)**, the HPLC chromatogram of standard substance **(B)**. 1.8-epi-loganic acid, 5. Plantamajoside, 8. Acteoside, 9. Luteoloside, 11. Astragalin, 13. Tricin 7-O-β-d-glucopyranoside, 16. Luteolin, 19. Chrysoeriol, 20. Tricin.

420 g of *Lagos brachystachya* Maxim was dried and cut into pieces and then heated and extracted under reflux with 10 times the volume of 75% ethanol three times. The extract was filtered, and the ethanol was recycled under reduced pressure by a rotary evaporator and concentrated to obtain ELB (35.67 of yield). The qualitative analysis of components in ELB was employed by liquid chromatography-tandem mass spectrometry (LC-MS/MS). The liquid chromatography equipment consists of a Shimadzu LC-20AD pump and Shimadzu SPD-20A detector. Furthermore, the accurate mass spectrometric analysis experiments were performed in negative ion mode on a TripleTOF 5600 + system with an electrospray ion source (AB Sciex, California, United States). Analysis was carried out on a C_18_ column (2.1 × 100 mm, 1.8 µm), and the column temperature was maintained at 35°C. The mobile phase was composed of A (0.1% formic acid in water, *v*/*v*) and B (acetonitrile) using a 10–35% B gradient elution at 0–48min 35–95%B at 48–55 min. The injection volume for the sample was 2 μL. The flow rate was 0.25 ml/min. The operation parameters were as follows: curtain gas, 40 psi; ion source gas 1 and ion source gas 2,50 psi; ion spray voltage floating, 4,500 V; temperature, 500°C; collision energy, −35eV; and collision energy spread, 10 eV. Data were managed with PeakView software (AB Sciex, California, United States). MS^2^ fingerprints of components in ELB were referred to databases like Metlin, ChemSpider, and MassBank and some related references for preliminary confirmation. Qualitative analysis of the above components of ELB was performed using high-performance liquid chromatography (HPLC) with Shimadzu SPD-20A detector using the standard substance. Analysis was carried out on an InertSustain C_18_ column (250 mm × 4.6 mm, 5 μm), and the column temperature was maintained at 30°C. The mobile phase was composed of A (0.1% formic acid in water, *v*/*v*) and B (acetonitrile) using a gradient elution of 5–10% B at 0–5 min, 10–20% B at 5–40 min, 20–22% B at 40–50 min, 22–35% B at 50–60 min, and 35–90% B at 60–85 min. The flow rate was set at 1.0 ml/min, the injection volume was 10 μL, and the monitoring wavelength was 256 nm. 8-epi-loganic acid (1.91 mg), acteoside (2.04 mg), luteoloside (1.21 mg), astragalin (1.75 mg), luteolin (1.09 mg), chrysoperiol (1.15 mg), tricin (1.15 mg), tricin 7-O-β-d-glucopyranoside (1.46 mg) were weighed individually. Then respectively dilute to 5 ml volumetric flask with methanol to obtain stock solutions of each reference substance, accurately weigh plantamajoside (1.65 mg) into 10 ml volumetric flask, measuring 200 µL from each stock solution of a reference substance, adding into 10 ml volumetric flask with plantamajoside, dilute to 10 ml with methanol, passing through 0.22 µm microporous membrane, collecting filtrate for analysis, tricin (no. PS011200), chrysoperiol (no. PS012019), astragalin (no. PS011379) were purchased from Chengdu Pusi Biotechnology Co., Ltd. (Chengdu, P. R. China). Luteolin (no. PRF10052843), Luteoloside (no. PRF10092803), Acteoside (no. PRF10062801) were purchased from Chengdu Purufa Technology Development Co., Ltd. (Chengdu, P. R. China), with purity ≧ 98%. 8-epi-loganic acid and tricin 7-O-β-d-glucopyranoside were prepared by our laboratory (purity was ≧ 95%). The HPLC fingerprint of ELB is provided in Figure 1.

### Molecular docking

The 3D compound structure of the compounds from ELB was downloaded from TCMSP and PubChem. Crystal TLR4 (3FXI) structure was downloaded from RCSB. Autodock Vina was used for molecular docking. The complex was then analyzed by Pymol.

### Animal modeling and drug administration

A total of 70 rats were randomly divided into seven groups, with 10 rats in each group: control group, model group (chronic alcoholic liver injury combined with gouty arthritis), colchicine group (0.3 mg/kg), Bifendate group (100 mg/kg), and ELB (2.0, 1.0, and 0.5 g/kg) (Shan et al., 2020b). All the groups except the control group were orally administrated with edible alcohol for 8 consecutive weeks. The dosage was 4 ml/kg in the first week, and then increased by 2 ml/kg every week to 10 ml/kg and maintained for 5 weeks. Colchicine, Bifendate, or ELB was orally administrated 6 h before alcohol consumption. The control and model groups were orally administrated with saline. The administrated volume was 10 ml/kg. At the end of the experiment, the volume of the right posterior toe was measured before administration. The inclined surface of the needle port of No.6 penetrated the right ankle cavity at an angle of 45 with the tibia towards the anterior superior. The rat model of acute gouty arthritis was established by injecting 0.1 ml of monosodium urate (MSU) solution of concentration at 50 mg/ml. The same parts of the ankle joints of rats were measured by toe volume meter 4, 6, 8, 12, 24, and 48 h after MSU injection. After the last administration, rats were deprived of food for 12 h and then anesthetized with 10% chloral hydrate (3 ml/kg). Then blood was collected from the abdominal aorta. The same parts of the rat liver and joint synovial membrane in each group were fixed in 4% paraformaldehyde. The rest of the tissues were stored at −80°C refrigerators for further measurement.

### Detection of ankle swelling

The volume of the right posterior ankle was measured with a toe volume meter before MSU injection and then measured at each time point (4, 6, 8, 12, 24, and 48 h) after MSU injection. The swelling degree of the rat ankle was calculated according to the formula: joint swelling degree = (joint volume at the measurement point—initial volume)/initial volume ×100%.

### Measurement of serum index of alcoholic liver injury and gouty arthritis

The contents of serum UA, XOD, IL-1β, IL-6, TNF-α, ALT, AST, TC, TG, ALP, AKP, and TBIT and the contents of hepatic SOD and MDA, were measured according to the commercial kits.

### HE staining

The liver and synovial tissues fixed with 4% paraformaldehyde were taken and trimmed with a blade in an embedding box. The tissue was rinsed overnight with running water, and then subjected to the conventional dehydration treatment from low to the high concentration of ethanol (50, 75, 85, 95%, anhydrous ethanol, and xylene). Subsequently, paraffin wax dipping, embedding, slicing, HE staining, and pathological morphological changes were observed under the microscope. The scoring criteria were indicated in Table 1.

**TABLE 1:** Histopathological scoring criteria of liver tissue and synovial membrane of alcoholic liver injury combined with gouty arthritis.

liver tissue	Synovial tissue	Score
The cell structure was normal, and there was no inflammatory cell infiltration	Synovial tissue with smooth edges and no inflammatory cells	0
Cell structure is normal, with a small amount of inflammatory cell infiltration	A small number of inflammatory cells	1
Cell structure without obvious abnormalities, a large number of inflammatory cell infiltration	obvious vascular congestion	2
Fat vacuoles	A large number of inflammatory cells, local inflammatory cell infiltration	3
Clusters of inflammatory cell infiltration and a large number of fat vacuoles were recorded	Inflammatory cells were gathered in clusters, and the cells were scattered	4
The structures of hepatic lobules were unclear and the nuclei disappeared	Synovial hyperplasia and tissue necrosis appeared	5

### Western blot

Liver and synovial tissues were placed in a 2 ml centrifuge tube and added 2%SDS lysis solution with proteases and phosphatase inhibitors. The tissue was ground by a tissue homogenizer and set at room temperature for 20 min to lyse it fully. The tissues were centrifuged at 12,000 g for 15 min at 4°C. The protein concentration of the collected supernatant was determined by a BCA kit. The samples were collected and denatured at 100°C for 5 min. Samples were prepared according to the concentration and subjected to routine loading electrophoresis. After electrophoresis, the gel was transferred to the PVDF membrane at 250 mΑ 90 min. The PVDF membrane was blocked at room temperature for 2 h in 5% of defatted milk. The membrane was washed three times with TBST, 10 min each time, and the primary antibody was incubated at 4°C overnight. The secondary antibody was incubated for 1 h on a horizontal shaking table the next day. ECL luminescent liquid was used for color development, and the gray values of each band were calculated by Image Lab software.

### Statistical analysis

The results were expressed by means ± SD. Gray values of protein bands were scanned by Image Lab software, and then data were processed by GraphPad Prism 7. One-way ANOVA followed Dunnett’s multivariate analysis was used to analyze the differences between groups. Statistical significance was considered as *p* < 0.05.

## Results

### Effect of extracts of *Lagotis brachystachya* on ankle swelling in rats with gouty arthritis

**FIGURE 2:**
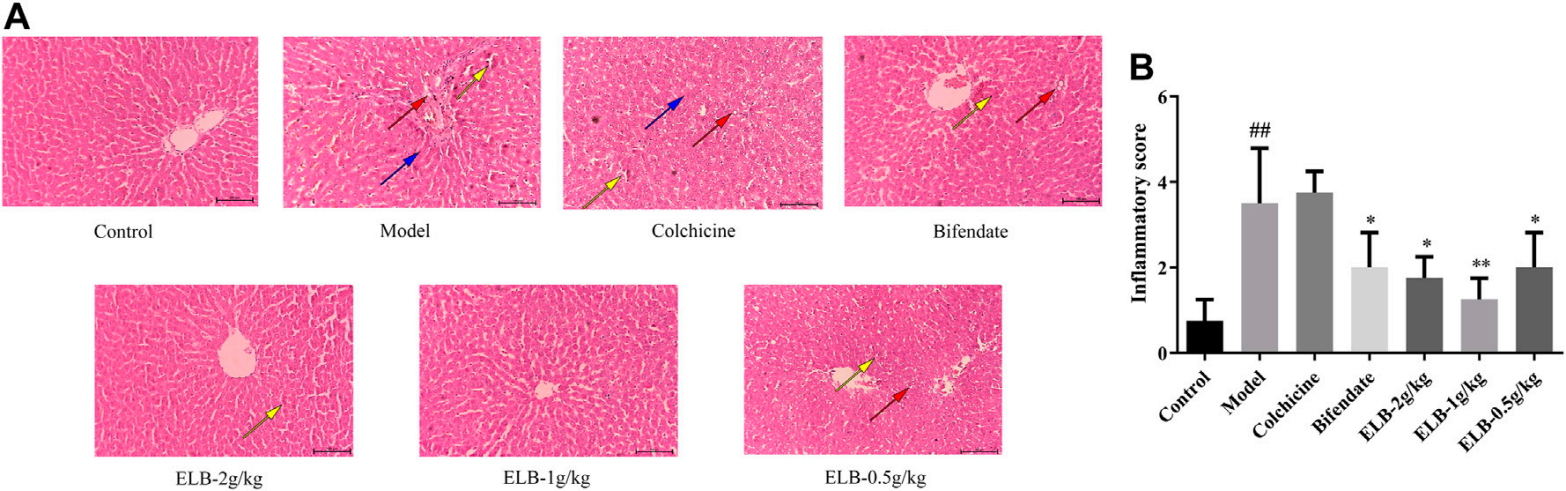
Histopathological evaluation showing the effects of ELB on liver tissue in rats with chronic alcoholic liver injury and gouty arthritis. Representative images **(A)**. Histopathological quantitative score **(B)**. The red arrow indicates fat vacuoles, yellow arrow indicates eosinophilic degeneration, blue arrow indicates disappearance of the nucleus.

As shown in Figure 2, there was no significant change in the degree of ankle swelling in the control group at each time point after the administration with the sterile saline. Compared with the control group, the ankle joints of rats in the model group exhibited noticeable swelling at each time point (*p* < 0.05, *p* < 0.01), which peaked at 12 h after modeling and then presented a slight downward trend. Compared with the model group, 48 h after modeling, the positive drug colchicine group and Bifendate group, ELB at high, medium, and low dose group, showed a significant downward trend.

### Effects of extracts of *Lagotis brachystachya* on serum TNF-α, IL-1β, and IL-6 contents in rats with chronic alcoholic liver injury and gouty arthritis

As shown in Table 2, compared with the control group, serum TNF-α, IL-1β, and IL-6 levels in the model group were significantly increased (*p* < 0.01). Compared with the model group, the levels of IL-6 were significantly reduced in the Bifendate group (*p* < 0.05). The serum levels of TNF-α were reduced considerably in the ELB high dose group (*p* < 0.05). The serum levels of TNF-α, IL-1β, and IL-6 were significantly reduced in the ELB medium-dose group (*p* < 0.05, *p* < 0.01). The serum levels of TNF-α were significantly decreased in the low-dose ELB group (*p* < 0.05).

**TABLE 2:** Effects of total extract of *Lagotis brachystachya* on serum TNF-α, IL-1β, and IL-6 of rats with chronic alcoholic liver injury and gouty arthritis (x ± *s*, *n* = 8).

Group	Dosage (g·kg^−1^)	TNF-α(pg/ml)	IL-1β(pg/ml)	IL-6 (pg/ml)
Control	—	47.54 ± 29.56	85.45 ± 42.90	58.49 ± 17.27
Model	—	125.97 ± 63.37^##^	200.24 ± 48.76^##^	125.41 ± 34.47^##^
Colchicine	0.0003	94.79 ± 48.00	137.43 ± 45.19	89.35 ± 33.23
Bifendate	0.1	74.85 ± 38.76	132.52 ± 65.56	84.22 ± 26.18*
Lagotis brachystachya	2.0	61.55 ± 27.67*	143.42 ± 54.73	95.95 ± 18.68
Lagotis brachystachya	1.0	57.25 ± 32.12*	110.47 ± 60.03*	69.86 ± 12.53**
Lagotis brachystachya	0.5	51.96 ± 31.20*	126.12 ± 56.79	98.45 ± 12.87

compared with control group, ^# #^ *p* < 0.01; Compared with the model group ^*^ *p* < 0.05, ^* *^ *p* < 0.01.

### Effect of extracts of *Lagotis brachystachya* on serum ALT, AST, TC, TG, AKP, TBIL, UA, and XOD of rats with chronic alcoholic liver injury and gouty arthritis

As shown in Table 3, compared with the control group, ALT, AST, TC, TG, AKP, TBIL, UA, and XOD in the model group were significantly increased (*p* < 0.01). The levels of ALT, AST, TBIL, and XOD in the colchicine group were significantly increased (*p* < 0.05, *p* < 0.01). UA level in the Bifendate group was significantly increased (*p* < 0.05). Compared with the model group, the AKP level in the colchicine group was significantly down-regulated (*p* < 0.01). The levels of ALT, TC, AKP, TBIL, and XODin the Bifendate group were significantly down-regulated (*p* < 0.05, *p* < 0.01). ALT, TC, TBIL, and XOD levels were significantly down-regulated in the ELB high dose group (*p* < 0.05, *p* < 0.01). ALT, AST, TC, TG, AKP, TBIL, XOD, and UA levels were significantly down-regulated in the ELB medium-dose group (*p* < 0.05, *p* < 0.01). ALT, AST, TC, AKP, TBIL, and UA levels were significantly down-regulated in the ELB low dose group (*p* < 0.05, *p* < 0.01).

**TABLE 3:** Effects of total extract of *Lagotis brachystachya* on serum ALT, AST, TC, TG, AKP, TBIL, XOD, and UA protein of rats with chronic alcoholic liver injury and gouty arthritis (x ± *s*, *n* = 8).

Group	Dosage (g·kg^−1^)	ALT (U·L^−1^)	AST (U·L^−1^)	TC (mmol·L^−1^)	TG (mmol·L^−1^)	#0070C0; ALP(U· L^−1^)	TBIL (µmol·L^−1^)	XOD (U·L^−1^)	UA (µmol·L^−1^)
Control	—	11.76 ± 1.66	13.24 ± 3.47	1.68 ± 0.58	0.74 ± 0.15	166.27 ± 19.16	3.69 ± 0.82	19.87 ± 2.00	58.33 ± 6.38
Model	—	20.49 ± 2.63^##^	17.80 ± 1.75^#^	2.49 ± 0.48^#^	1.40 ± 0.27^##^	266.89 ± 33.30^##^	7.09 ± 1.38^##^	32.18 ± 4.89^##^	77.60 ± 13.04^##^
Colchicine	0.0003	18.92 ± 8.51^##^	17.75 ± 2.75^#^	2.10 ± 0.45	1.21 ± 0.56	191.95 ± 29.82^**^	5.85 ± 1.72^#^	31.44 ± 6.24^##^	61.11 ± 11.96
Bifendate	0.1	13.12 ± 1.32^**^	14.61 ± 3.79	1.61 ± 0.59^**^	0.96 ± 0.24	189.13 ± 43.80^**^	3.88 ± 1.05^**^	25.66 ± 4.55^*^	72.97 ± 4.77^#^
Lagotis brachystachya	2.0	12.86 ± 1.40^**^	12.78 ± 1.99	1.70 ± 0.28^*^	1.01 ± 0.29	224.96 ± 21.4	3.78 ± 1.44^**^	22.57 ± 2.52^**^	63.46 ± 10.57
Lagotis brachystachya	1.0	12.49 ± 2.03^**^	11.85 ± 2.43^*^	1.78 ± 0.33^*^	0.86 ± 0.13^*^	132.88 ± 29.17^**^	3.65 ± 1.09^**^	19.01 ± 2.13^**^	56.73 ± 7.90^**^
Lagotis brachystachya	0.5	13.41 ± 1.79^**^	14.16 ± 2.75^**^	1.72 ± 0.22^*^	0.99 ± 0.27	187.43 ± 34.79^**^	4.35 ± 1.60^**^	26.57 ± 3.93	62.07 ± 6.13^*^

compared with control group, ^#^ *p* < 0.05, ^# #^ *p* < 0.01; Compared with the model group ^*^ *p* < 0.05, ^* *^ *p* < 0.01.

### Effects of extracts of *Lagotis brachystachya* on superoxide dismutase and malondialdehyde contents in liver homogenate of rats with chronic alcoholic liver injury and gouty arthritis

As shown in Table 4, compared with the control group, SOD levels in the model and colchicine groups were significantly reduced, and MDA levels were significantly increased. Compared with the model group, Bifendate significantly decreased MDA levels, ELB at high dose and medium-dose significantly increased SOD, but decreased MDA levels.

**TABLE 4:** Effects of total extract of *Lagotis brachystachya* on the contents of SOD and MDA in the liver homogenate of the rat model with chronic alcoholic liver injury and gouty arthritis (x ± *s*, n = 8).

Group	Dosage (g·kg^−1^)	SOD (U/mg protein)	MDA (nmol/mg protein)
Control	—	390.21 ± 35.67	2.59 ± 0.91
Model	—	276.49 ± 54.16^##^	4.30 ± 1.21^##^
Colchicine	0.0003	241.66 ± 53.66^##^	4.02 ± 0.47^#^
Bifendate	0.1	326.55 ± 57.87	2.51 ± 0.73^**^
Lagotis brachystachya	2.0	353.97 ± 50.32*	3.80 ± 0.97
Lagotis brachystachya	1.0	384.19 ± 17.87**	2.89 ± 0.84^*^
Lagotis brachystachya	0.5	345.31 ± 43.65	2.96 ± 0.33^*^

compared with control group, ^#^ *p* < 0.05, ^# #^ *p* < 0.01; Compared with the model group ^*^ *p* < 0.05, ^* *^ *p* < 0.01.

### Effects of extracts of *Lagotis brachystachya* on liver morphology in rats with chronic alcoholic liver injury and gouty arthritis

**FIGURE 3:**
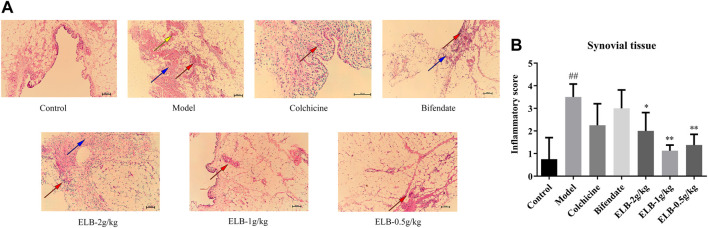
Histopathological evaluation showing the effects of ELB on synovial tissue in rats with chronic alcoholic liver injury and gouty arthritis. Representative images **(A)**. Histopathological quantitative score **(B)**. The red arrow indicates eosinophilic degeneration, yellow arrow indicates obvious vascular congestion, blue arrow indicates the scattered cell arrangement.

As shown in Figure 3A, the morphology of hepatocytes in the liver tissue of control rats was controlled, the hepatic sinusoids and portal areas were clear, and the hepatocytes were arranged in a divergent shape toward each other. The hepatocytes in the model group were disorganized and bulky. A large number of lipid droplet vacuoles formed, accompanied by inflammatory infiltration, and hepatocytes showed signs of focal necrosis. Compared with the model group, the hepatocytes were arranged neatly in Bifendate group and ELB low-dose group. The inflammatory infiltration was reduced, with small lipid droplet vacuoles remaining. Under the microscope, the situation was improved in the medium and high dose groups of ELB. The hepatocytes were arranged neatly and dispersed to all sides, and the cell edema and inflammatory infiltration were significantly alleviated. According to Figure 3B, the histopathological score in model group was significantly higher than that in control group. In contrast, administration with Bifendate and ELB decreased the histopathological score.

### Effects of extracts of *Lagotis brachystachya* on synovial morphology in rats with chronic alcoholic liver injury and gouty arthritis

**FIGURE 4:**
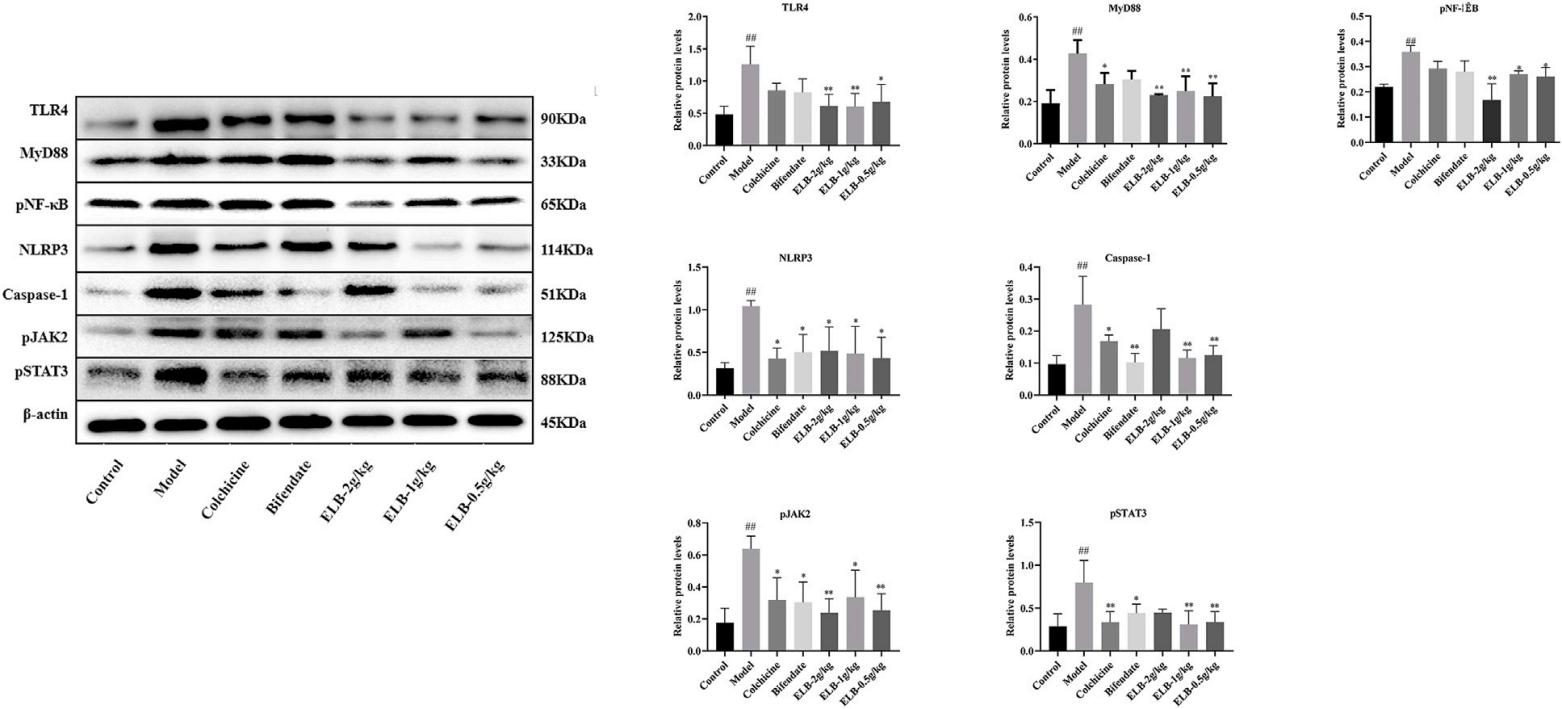
Effects of ELB on protein levels of TLR4, MyD88, pNF-κB, NLRP3, caspase-1, pJAK2, and pSTAT3 in the liver of a rat model with chronic alcoholic liver injury and gouty arthritis. Note: compared with control group, ^#^ *p* < 0.05, ^# #^ *p* < 0.01; Compared with the model group ^*^ *p* < 0.05, ^* *^ *p* < 0.01.

As shown in Figure 4A, the synovial cells of control rats had complete morphology, smooth edges, and no protrusions, which were arranged neatly and orderly. No inflammatory cell infiltration was observed. Compared with the control group, the synovial cells in the model and Bifendate groups had incomplete morphology, broken edges and disorderly arrangement, prominent inflammatory cell infiltration, and apparent vascular congestion. The infiltration state of inflammatory cells was improved in the colchicine group and the ELB at high, medium, and low doses. The state was the best in the colchicine group and the medium-dose group. According to Figure 4B, the histopathological score in model group was significantly higher than that in control group. However, this alteration was reversed by ELB.

### Effects of extracts of *Lagotis brachystachya* on the activity of TLR4/MyD88/NF-κB, NLRP3, and JAK2-STAT3 signaling pathway proteins in the hepatic tissue

As shown in Figure 4, compared with the control group, the protein levels of TLR4, MyD88, pNF-κB, NLRP3, Caspase-1, pJAK2, and pSTAT3 in the model group were significantly increased (*p* < 0.05. *p* < 0.01). Compared with the model group, the levels of NLRP3, caspase-1, and pSTAT3 proteins in the colchicine group were significantly down-regulated (*p* < 0.05). Caspase-1 and pJAK2 proteins in the Bifendate group were significantly down-regulated (*p* < 0.05). The protein levels of TLR4, MyD88, pNF-κB, and pJAK2 in the ELB high dose group were significantly down-regulated (*p* < 0.05. *p* < 0.01). The protein levels of TLR4, MyD88, Caspase-1, and pSTAT3 in the ELB medium-dose group were significantly down-regulated (*p* < 0.05. *p* < 0.01). The protein levels of TLR4, MyD88, NLRP3, Caspase-1, pJAK2, and pSTAT3 in the ELB low dose group were significantly down-regulated (*p* < 0.05. *p* < 0.01).

### Effects of extracts of *Lagotis brachystachya* on the activity of TLR/MyD88/NF-κB, NLRP3, and JAK2-STAT3 signaling pathway proteins in synovial tissue

**FIGURE 5:**
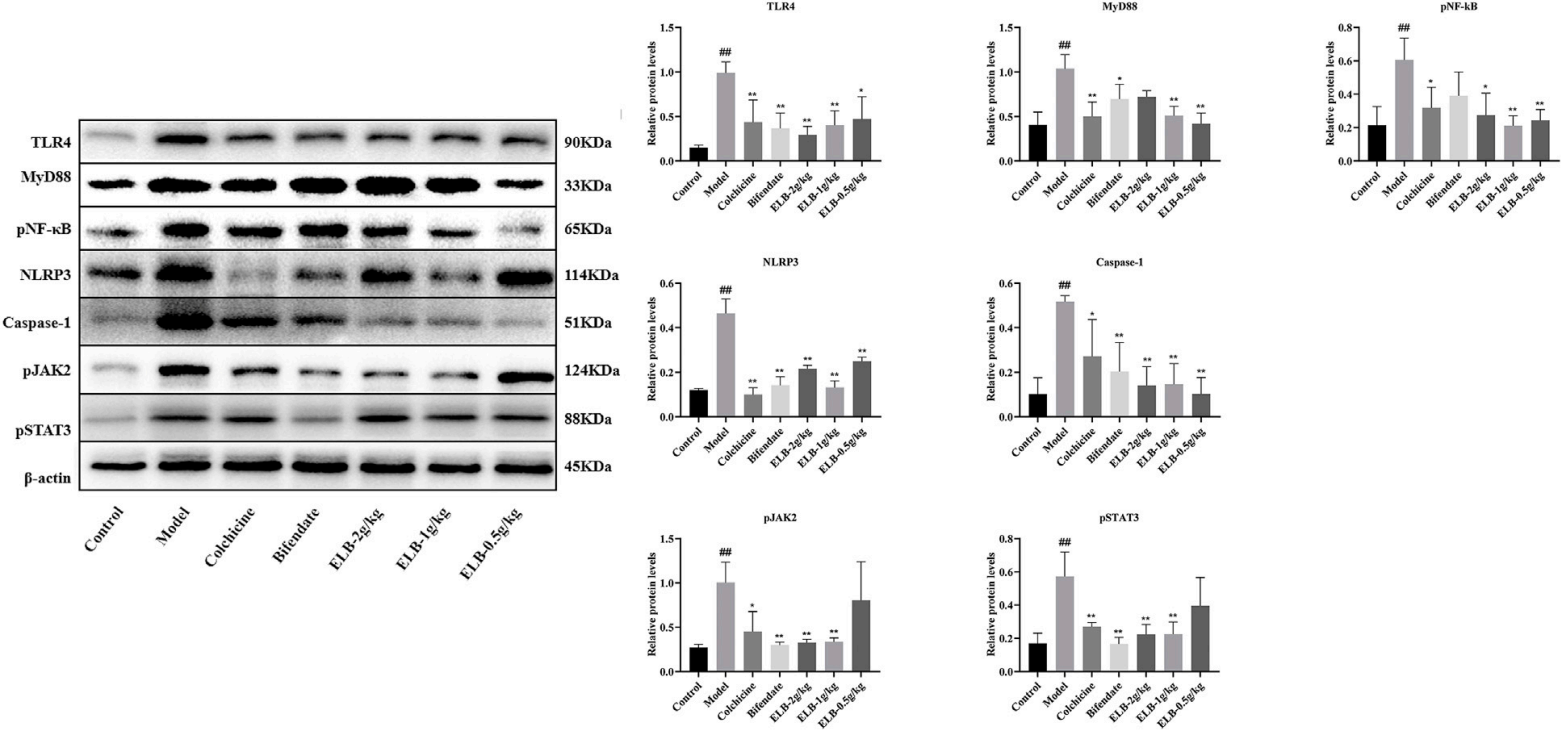
Effects of ELB on protein levels of TLR4, MyD88, pNF-κB, NLRP3, caspase-1, pJAK2, and pSTAT3 in the synovial tissue of a rat model with chronic alcoholic liver injury and gouty arthritis. Note: compared with control group, ^#^ *p* < 0.05, ^# #^ *p* < 0.01; Compared with the model group ^*^ *p* < 0.05, ^**^ *p* < 0.01.

As shown in Figure 5, compared with the control group, the protein levels of TLR4, MyD88, pNF-κB, NLRP3, Caspase-1, pJAK2, and pSTAT3 in the model group were significantly increased (*p* < 0.05. *p* < 0.01). The levels of Caspase-1 protein in the colchicine group were significantly increased (*p* < 0.05). Compared with the model group, the protein levels of colchicine TLR4, MyD88, NLRP3, and pSTAT3 were significantly down-regulated (*p* < 0.05). The protein levels of TLR4, NLRP3, Caspase-1, pJAK2, and pSTAT3 in the Bifendate group were significantly down-regulated (*p* < 0.05). The levels of TLR4, pNF-κB, NLRP3, Caspase-1, pJAK2, and pSTAT3 proteins in the ELB high dose group were significantly down-regulated (*p* < 0.05. *p* < 0.01). The protein levels of TLR4, MyD88, pNF-κB, NLRP3, Caspase-1, pJAK2, and pSTAT3 in the ELB medium-dose group were significantly down-regulated (*p* < 0.05. *p* < 0.01). The levels of TLR4, MyD88, pNF-κB, NLRP3, and Caspase-1 proteins in the ELB low dose group were significantly down-regulated (*p* < 0.05. *p* < 0.01).

### The putative binding activity between chemical components of extracts of *Lagotis brachystachya* and TLR

**FIGURE 6:**
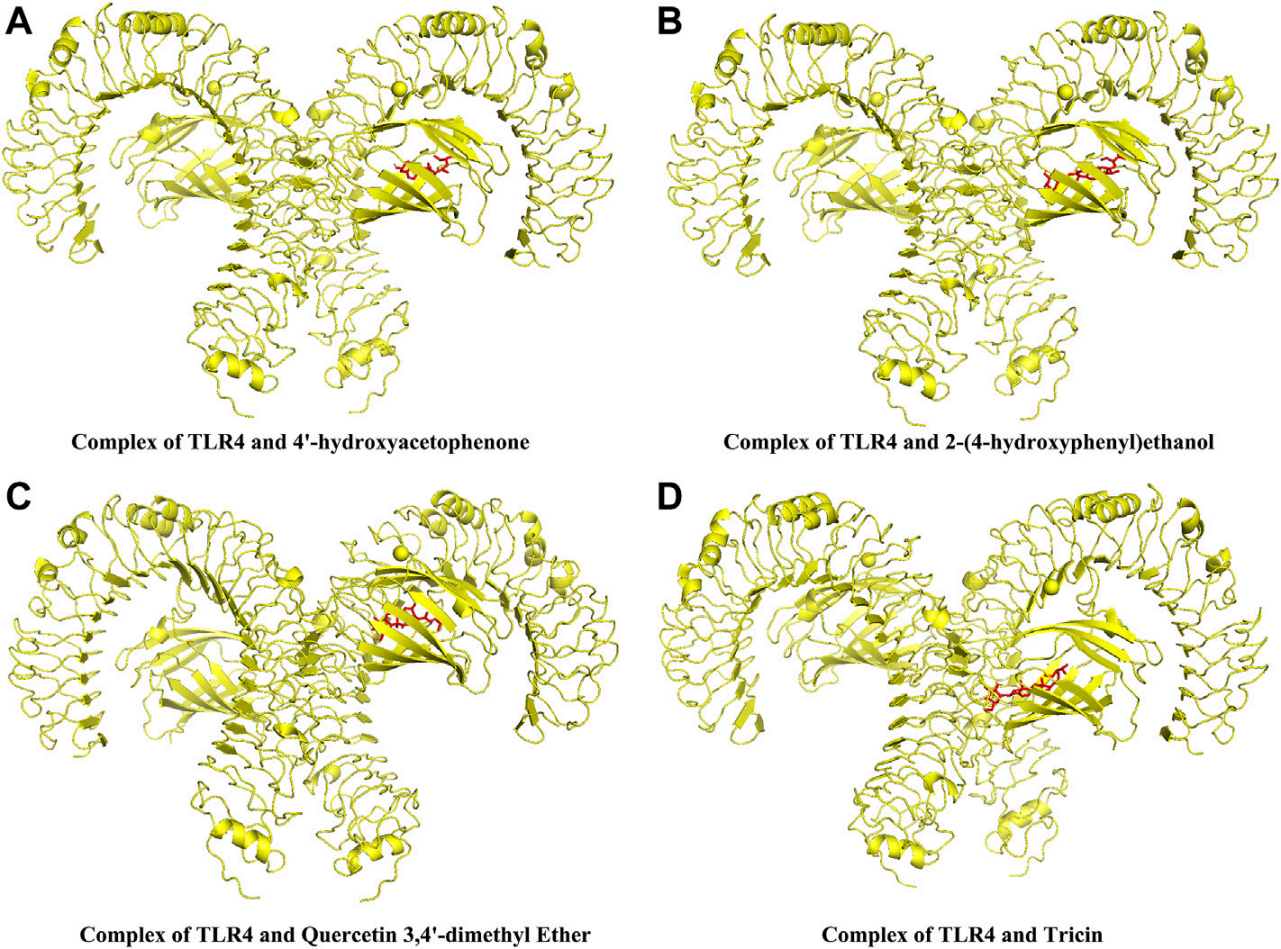
The docking complex between the chemical components of ELB and TLR4. The complexes of the top 4 highest scorings were shown. Complex of TLR4 and 4′-hydroxyacetophenone **(A)**. Complex of TLR4 and 2-(4-hydroxyphenyl)ethanol **(B)**. Complex of TLR4 and Quercetin 3.4′-dimethyl Ether **(C)**. Complex of TLR4 and Tricin **(D)**.

The affinity between chemical components of ELB and TLR4 was shown in Supplementary Table S1. The complex with top 4 binding affinity was shown in Figure 6. The docking results showed that 4′-hydroxyacetophenone, 2-(4-hydroxyphenyl)ethanol, Quercetin 3.4′-dimethyl Ether, and Tricin can embed inside TLR4, thus preventing the putative binding between TLR4 and its activators. These observations predict that 4′-hydroxyacetophenone, 2-(4-hydroxyphenyl)ethanol, Quercetin 3.4′-dimethyl Ether, and Tricin might be the active components for the inhibition of TLR/MyD88/NF-κB signaling.

## Discussion

The pathogenesis of alcoholic liver injury and gouty arthritis is complex and still unclear. Although we have made great efforts in basic research and clinical practice in recent decades, the treatment efficacy is still unsatisfactory. In this respect, it is an urge to elucidate other new targets for treating alcoholic liver injury and gouty arthritis. Inflammation plays an important role in the development of alcoholic liver injury (Liu Q. et al., 2021). Anti-inflammation is a new therapeutic target for alcoholic liver injury involving TLR and other signaling pathways. The activation of NF-κB and STAT leads to the increased release of inflammatory cytokines (Wen et al., 2021a; Wen et al., 2021b; Zhang et al., 2022). Clinical observation has demonstrated that acute gout attacks often accompany the alcoholic liver injury. The onset of many gout patients is closely related to their drinking behavior. After ingesting alcohol, urate will be produced through the liver and rapidly increase serum uric acid (Dalbeth et al., 2016).

The biochemical analysis found that ALT, AST, TC, TG, AKP, TBIT, XOD, UA, TNF-α, IL-1β, and IL-6 in the serum of rats in the model group were significantly increased. Meanwhile, hepatic SOD levels were significantly decreased, and hepatic MDA levels were significantly increased, indicating that the two diseases were successfully replicated in rats. Compared with the model group, ELB could reverse the abnormal alteration in the serum and liver. On the other hand, the pathological evaluation showed that the hepatocytes of each dose group of ELB were arranged neatly, and the inflammatory infiltration and fat vacuoles were reduced. The edges of synovial tissues were neat and smooth, with complete cell morphology, reduced inflammatory cell infiltration, and improved vascular congestion. These results indicated that ELB could alleviate both the symptoms of alcoholic liver injury and gouty arthritis.

NF-κB is widely involved in various pathophysiological processes of the body by regulating gene transcription and expression, such as defense responses to pathogens or other damages, tissue damage and stress responses, and promotion of cell differentiation and apoptosis. The activation of NF-κB is closely related to alcoholic liver injury and gouty arthritis (Li et al., 2019; Liu et al., 2022). Accumulating studies have also confirmed that inhibiting the NF-κB signaling pathway can attenuate the symptoms of alcoholic liver injury and gouty arthritis by inhibiting the release of proinflammatory cytokines (Amen et al., 2020; Ouyang et al., 2021). In the present study, ELB could significantly inhibit TLR4/NF-κB/MyD88 signaling pathway and proinflammatory cytokines in both the hepatic and synovial tissues, indicating that the therapeutical action of ELB is mediated by the inhibition of TLR4/NF-κB/MyD88/cytokine pathway.

The NLRP3 inflammasome is the inflammasome with the most well-studied function at present. It is a kind of multi-protein complex that belongs to the intracellular pattern recognition receptor and can be expressed in various immune cells. During the inflammatory response of alcoholic liver injury and gouty arthritis, the uncontrolled IL-1β processed by NLRP3 causes the inflammatory response and induces the release of more proinflammatory cytokines and adhesion molecules. The inflammatory response in turn aggravates hepatic and synovial tissue inflammation (Dinarello, 2010; Shen et al., 2020). The NLRP3 levels of patients with alcoholic liver injury and gouty arthritis were significantly increased, indicating that NLRP3 inflammasome was activated in alcoholic liver injury and gouty arthritis (Wu et al., 2017; Chang et al., 2019). Similarly, the levels of IL-1β in plasma were positively correlated with the severity of alcoholic liver injury and gouty arthritis (Ilyas et al., 2019; Zhang et al., 2021). At the same time, NLRP3 inflammasome further aggravates alcoholic liver injury and gouty arthritis by activating plenty of macrophages. The present study found that the NLRP3/caspase-1 signaling pathway was reversed by administration with ELB. Similarly, recent studies have found that some natural products alleviated alcoholic liver injury and gouty arthritis by inhibiting NLRP3 inflammasome (Liu et al., 2019; Wang et al., 2021), which has guiding significance for the development and clinical application of alcoholic liver injury and gouty arthritis by targeting NLRP3.

It is well-known that proinflammatory factors could activate the JAK2/STAT3 signaling to aggravate the inflammatory response in hepatic and synovial tissues. This hyperactivated inflammatory signaling pathway has been demonstrated with liver fibrosis and swollen joints (Li et al., 2018; Xin et al., 2020). In the present study, we found that alcohol and MSU induced the JAK2/STAT3 signaling pathway activation accompanied by increased proinflammatory cytokines. On the contrary, ELB completely reversed the activation of JAK2/STAT3 signaling in both the hepatic and synovial tissues.

Finally, we used molecular docking to speculate the putative binding affinity between the active components of ELB and TLR4. The results indicated that 4′-hydroxyacetophenone, 2-(4-hydroxyphenyl)ethanol, Quercetin 3.4′-dimethyl Ether, and Tricin could bind with TLR4 have the strongest binding ability with TLR4, which indicated that 4′-hydroxyacetophenone, 2-(4-hydroxyphenyl)ethanol, Quercetin 3.4′-dimethyl Ether, and Tricin might be the active components for the inhibition of TLR/MyD88/NF-κB signaling. More detection is required to elucidate the target and mechanism of ELB in further study.

In conclusion, the current study indicates that ELB exhibits a protective effect in rats with chronic alcoholic liver injury and gouty arthritis, possibly mediated by inhibiting TLR4/MyD88/NF-κB, NLRP3, and JAK2-STAT3 signaling pathways in both the hepatic and synovial tissues. Thus, this study demonstrates that *Lagotis brachystachya* might be a promising candidate for treating inflammation-related liver injury and gouty arthritis.

## Data Availability

The original contributions presented in the study are included in the article/Supplementary Material, further inquiries can be directed to the corresponding author.
