# Supplementary figures and images for: Lagotis brachystachya maxim attenuates chronic alcoholic liver injury combined with gouty arthritis in rats via its anti-inflammatory activity
Source: Front Pharmacol. 2022 Sep 13;13:995777. doi: 10.3389/fphar.2022.995777 (PMC9513826; doi:10.3389/fphar.2022.995777)

## Synovial tissue HE staining in colchicine group

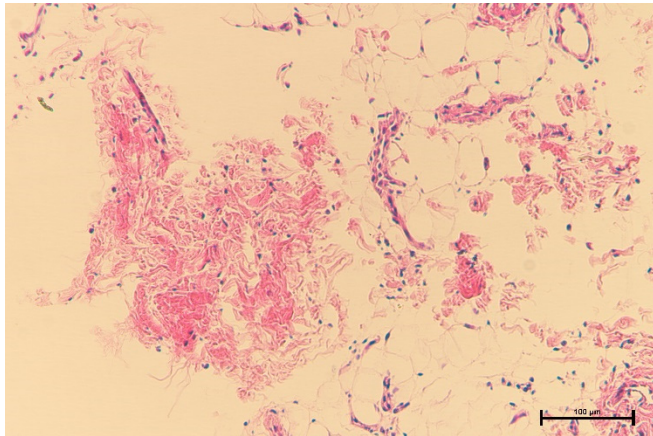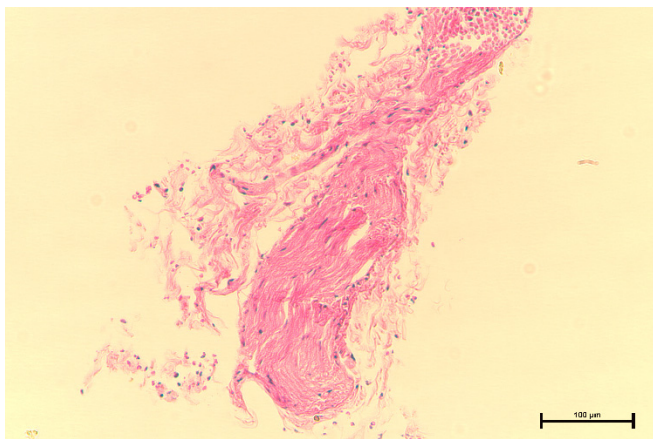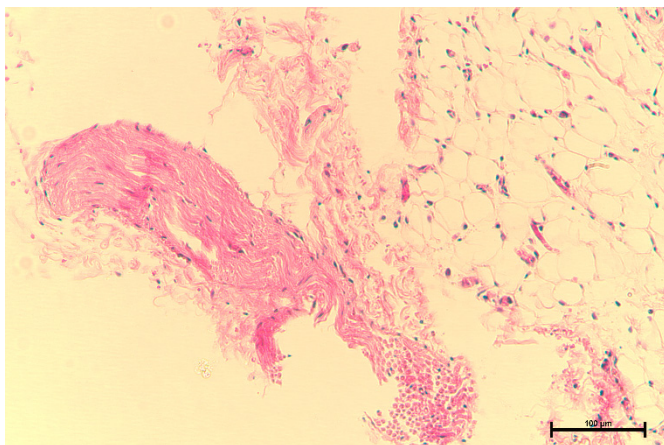

## Original MyD88 bands

### Liver

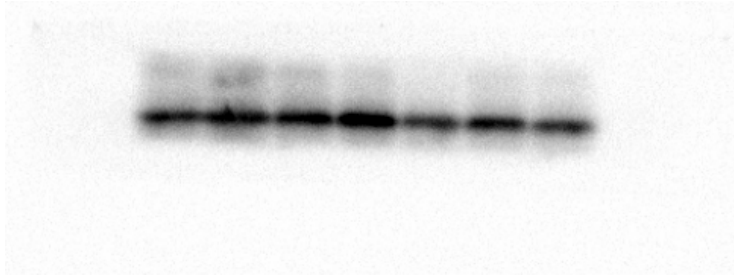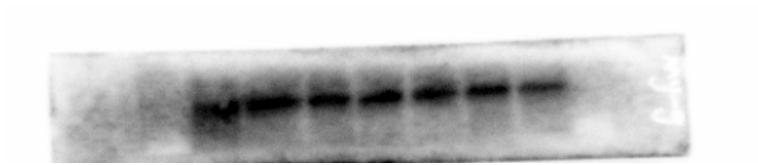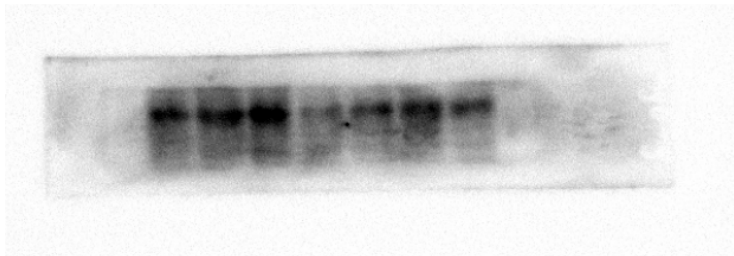

### Synovial

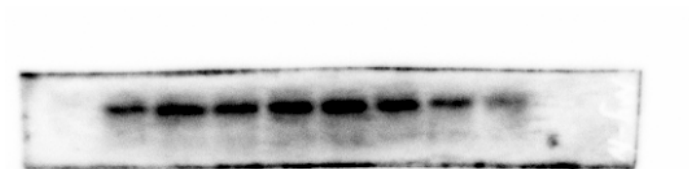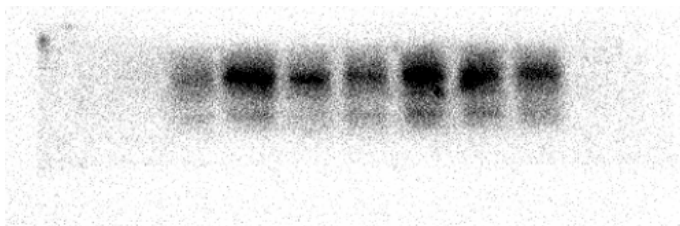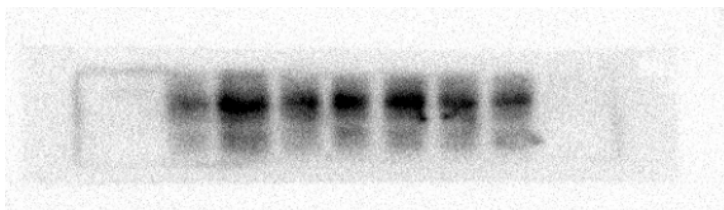

Supplement: Supplementary file 1 [file DataSheet1.PDF]
